# Foot tissue stress in chronic ankle instability during the stance phase of cutting

**DOI:** 10.1007/s11517-024-03276-9

**Published:** 2025-01-15

**Authors:** Peimin Yu, Xuanzhen Cen, Liangliang Xiang, Alan Wang, Yaodong Gu, Justin Fernandez

**Affiliations:** 1 Faculty of Sports Science, Ningbo University, Ningbo, China; 2 Auckland Bioengineering Institute, The University of Auckland, Auckland, New Zealand; 3 Department of Engineering Science and Biomedical Engineering, The University of Auckland, Auckland, New Zealand

**Keywords:** Free-form deformation, Lateral ankle sprain, Finite element analysis, Host mesh fitting, Personalized foot model

## Abstract

Lower limb biomechanics of chronic ankle instability (CAI) individuals has been widely investigated, but few have evaluated the internal foot mechanics in CAI. This study evaluated bone and soft tissue stress in CAI contrasted with copers and non-injured participants during a cutting task. Integrating scanned 3D foot shapes and free-form deformation, sixty-six personalized finite element foot models were developed. Computed Achilles tendon forces and measured regional plantar pressure were applied as boundary loading conditions for simulation. It was observed that the primary group differences in foot stress occurred during midstance and heel-off phases of the cutting task. Specifically, healthy individuals had significantly higher stress in the talus and soft tissue around the talus compared to CAI participants. In contrast, CAI participants had significantly higher stress in the cuneiforms and lateral forefoot bones during mid-stance and push-off phases. CAI participants appeared to adopt a protective strategy by transferring greater force to the lateral forefoot at the heel-off phase while lowering stress around the talus, which may be associated with pain relief near the ankle. These findings suggest further attention should be placed on internal stress in CAI at the push-off phase with implications for long-term foot adaptation.

**Graphical Abstract:**

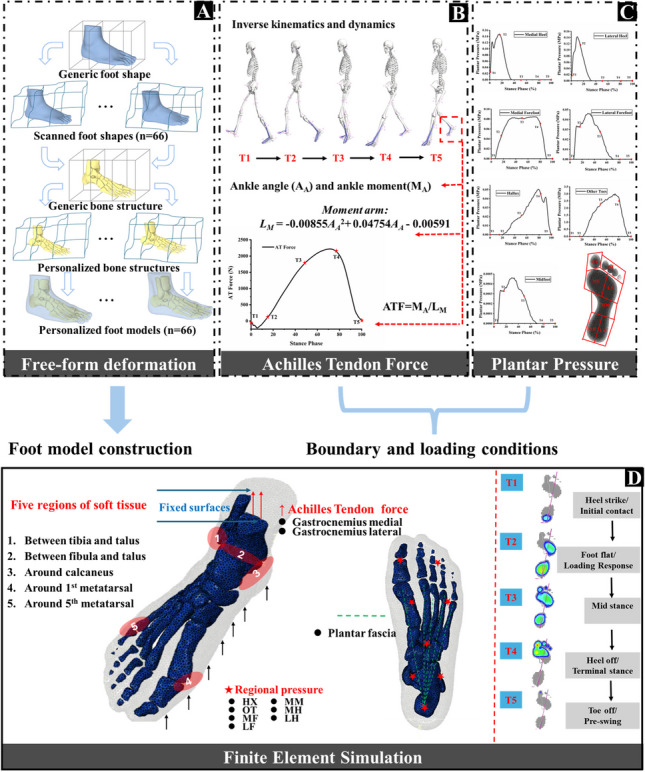

**Supplementary Information:**

The online version contains supplementary material available at 10.1007/s11517-024-03276-9.

## Introduction

Lateral ankle sprain (LAS) is one of the most common musculoskeletal injuries with a recurrent rate of approximately 40–73% [1–3]. Chronic ankle instability (CAI) describes the cluster of chronic symptoms that would develop after repetitive LAS, including the resulting sequela of pain, and instability often referred to as “giving way” in the literature [4]. Contrarily, some people who only sustain one incidence of LAS and are free from recurrent sprains can be classified as copers [4]. Hence, exploring the differences between these groups would help provide insight into potential mechanisms that are associated with repetitive LAS. Numerous studies have been conducted to compare the biomechanical differences in CAI during typical locomotive tasks including walking, running, and different types of landing tasks [5, 6]. However, little is known concerning the cutting maneuver, with this change-of-direction task often associated with a high risk of LAS [7].

Although many laboratory-based experiments have been conducted to reveal the biomechanical characteristics of CAI [5, 6, 8–11], there are few studies examining the internal tissue stresses in the foot. Finite element (FE) analysis may provide a quantitative evaluation tool for the internal mechanical response through simulation [12–14]. Several foot–ankle complex FE models have been developed to explore the influence of LAS [15–19]. Bae et al. [15] constructed the ankle FE model with different ligament-deficient and ligament-ruptured strategies to simulate the influence of ankle sprains during normal walking, finding the increased ankle joint contact pressures, strains, and translation during the push off phase at the ankle with lateral ligament injuries [15]. Considering that the inability of the ankle ligaments has been associated with talus rotational instability, Marta et al. [16] established the ankle FE model with pure internal talus rotations of different angles. With the angles of internal rotations of talus increased, the maximum contact pressure of the ankle cartilage increased. Novel contact regions in the anteromedial and posterolateral sections of the taler cartilage were observed [16]. To determine the role of lateral ankle ligaments on the syndesmotic stability, Mercan et al. developed ankle FE models with various ankle ligament injury models [17]. It was revealed that the anterior talofibular ligament injury may negatively influence syndesmotic stability. Furthermore, the combination of anterior talofibular ligament, anterior inferior talofibular ligament, and interosseous ligament injuries could lead to the greatest fibular translation [17]. Apart from the above studies, FE models have also been developed for the aim of clinical utilization [18, 19]. Wang et al. [18] constructed the foot model and lateral collateral ligament injury models to compare different surgical techniques for ligament reconstruction, finding that allogeneic tendon reconstruction might be the greater surgical technique for reconstructing lateral ankle ligament reconstruction without considering the complications. Wang and Cai [19] built two FE models of the initial contact phase and toe-off phase and found the center of pressure shifts in the anterolateral direction in ankle instability individuals, which could help quantitively monitor ligament injuries and rehabilitation progress [19]. FE models developed in these studies can roughly be divided into two types: (1) those including the intact ankle and (2) those imitating ankle injury. Limited by the number of FE models, no statistical analysis was conducted in those studies to compare the differences between healthy and injured joints. Furthermore, to the best of our knowledge, tissue stress characteristics in bony structures of CAI and copers have not been explored to date.

Generating FE meshes for each participant based on medical images typically requires manual manipulation and can be time-consuming. Free-form deformation (FFD), as a geometric morphing technique, enables users to build subject-specific shapes from template-based deformation [20]. It is achieved by deforming the lattice mesh which encloses the object of interest, followed by transforming the object within the bounding hull. The host-mesh variant of FFD involves fitting a host to data through transformations (Euclidean and affine operations), which in turn deform the enclosed object [21]. FFD has previously been applied in biomechanics and FE modeling by deforming generic atlas meshes to subject shapes including a 2-dimensional hand model and 3-dimensional femur bone and aorta [20]. Koh et al. used FFD to reconstruct 3-dimensional femur models through two X-ray images and three computed tomography images [22]. Furthermore, FFD has been used to improve hip joint center estimates [23]. In general, FFD has been presented in the literature as a time and cost-effective method for model reconstruction and may be useful in generating large populations of personalized FE models.

Therefore, the aim of the current study was to reconstruct personalized FE models based on FFD and statistically compare bone and soft tissue stress across CAI, copers, and healthy participants during the stance phase of a cutting maneuver. It is hypothesized that the primary stress difference among CAI, copers, and healthy individuals would be observed in the talus and fibula bones. Furthermore, given the directional shift of the cutting task, we also hypothesized altered stress characteristics in the metatarsal in CAI.

## Materials and methods

### Participants

Sixty-six male participants (22 CAI, 22 copers, and 22 healthy individuals) were recruited (Table 1) for the gait experiment after sample size calculation in G*Power (effect size = 0.4, α value = 0.05, power value = 0.8) [24, 25]. The selection criteria of CAI participants and copers were in agreement with the International Ankle Consortium guidelines and previous studies [4, 26, 27]. Gait experiments including capturing three-dimensional (3D) foot shapes, foot kinetics and kinematics, and foot pressure during the cutting task were based on our previous studies [27, 28]. Briefly, the involved limb for data collection in CAI group is the foot with Cumberland ankle instability tool (CAIT) score of 24 or lower. For copers, the involved limb was chosen as the foot with one incidence of LAS and CAIT score between 25 and 28. The dominant limb in the control group was selected as the involved limb. The left foot was mirrored in the medial–lateral direction to represent the pseudo-right foot for further process. Detailed inclusion criteria can be found in our previous studies [27, 28]. Ethics approval for this research was granted by the local ethics committee (The ethics approval number: UAHPEC21073).

**Table 1:** Demographical description of participants (mean ± standard deviation)

Variables	CAI	Coper	Control
*N*	22	22	22
Age, y	22.59 ± 1.92	22.45 ± 2.02	23.50 ± 2.50
Height, cm	182.30 ± 7.23	174.95 ± 5.08	177.64 ± 5.21
Weight, kg	79.02 ± 8.72	71.09 ± 8.05	73.98 ± 7.01
CAIT scores	19.63 ± 3.35	25.91 ± 0.81	29.63 ± 0.49

*CAI*, chronic ankle instability, *CAIT*, Cumberland Ankle Instability Tool

### Host-mesh free form deformation

**Fig. 1:**
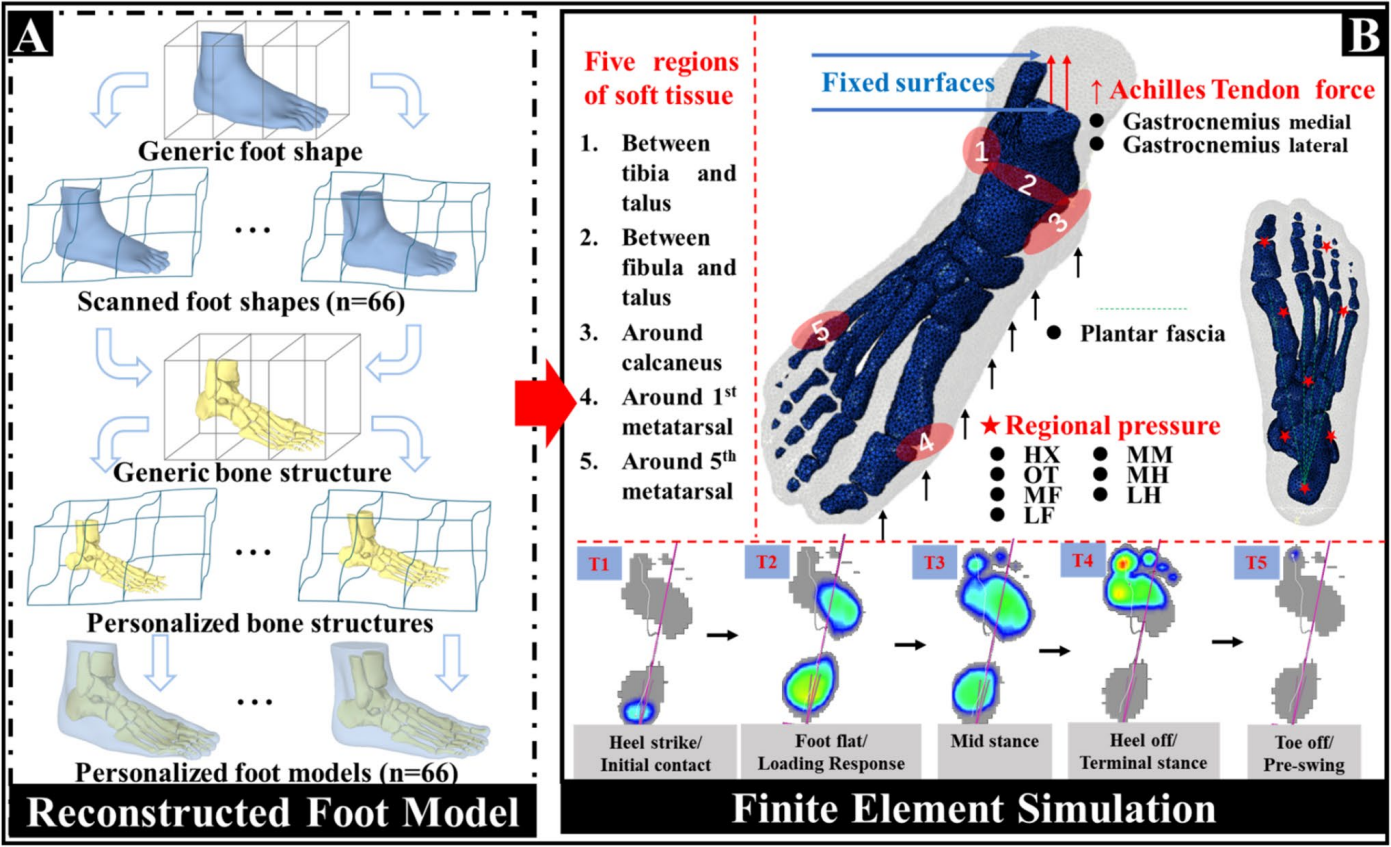
Illustration of the complete pipeline for personalized FE model development. **A** Personalized foot model reconstruction based on host-mesh free form deformation. **B** Applications of the boundary and loading conditions for finite element simulation

A generic foot model was reconstructed by the medical computer tomography (CT) images of the right foot of a healthy male participant (age 28 years, height 176 cm, mass 69 kg). An optime CT540 scanner (GE Healthcare, United States) was used to scan the foot under neutral and non-weight-bearing conditions with 0.625 mm between slices in the coronal plane. These images were then manually segmented in MIMICS v19.0 (Materialise Co., Ltd., Leuven, Belgium) to obtain the boundaries of bones and soft tissue. A previously established hybrid statistical morphometry free-form deformation framework [29] was used to generate internal bone structures of all participants based on 3D foot surface shapes, which is shown in Fig. 1A. In brief, the geometric alignment algorithm was applied to align the generic FE foot surface to the participant foot shape data captured by the Easy-Foot-Scanner (Kaunas, Lithuania). Following this, the registered FE foot surface was morphed using FFD to the target foot surface data. As part of this morphing, the internal foot structures were reconstructed. This process was repeated for 66 foot models. Details of the original free-form deformation implementation are given in [21] and validation of the technique for the foot presented in [29] with the technique able to reconstruct the foot shape of blinded data with the dice similarity coefficient (DICE) of 0.92 ± 0.01, and the internal bones with DICE of 0.84 ± 0.03.

### Model construction

**Fig. 2:**
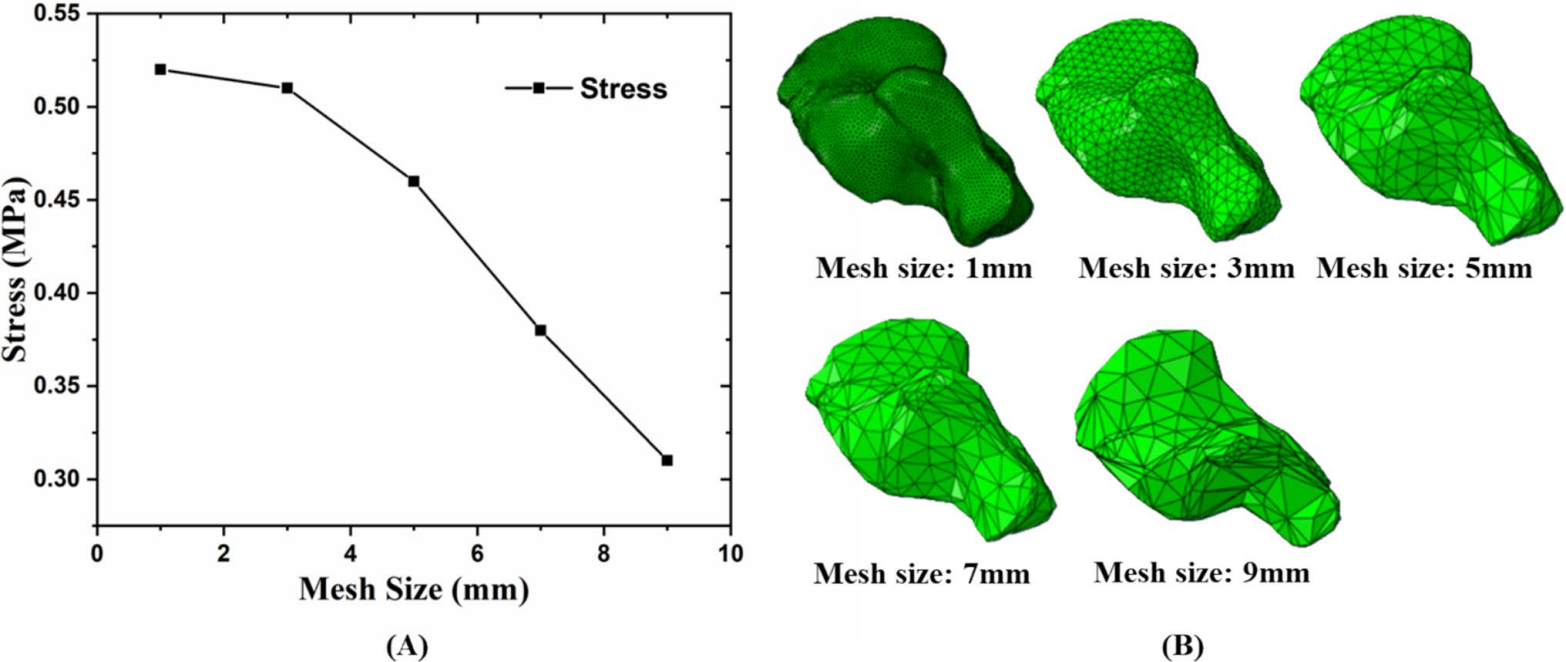
Pre-study mesh convergence analysis. Representative mesh convergence for calcaneus: results from different mesh sizes with same material properties and loading conditions (**A**) and different mesh sizes of calcaneus (**B**)

Geomagic Wrap 2021 (3D Systems. Rock Hill, USA) was used to generate STL formation point cloud data of the bone structures and to smooth both bone and soft tissue geometries. The geometries were then converted in Hypermesh 22.2 (Altair Company, Tory, MI, USA) to create solid mesh models. In terms of the soft tissue, volume Boolean operations were used to generate soft tissue volume, which subtracted bone structures from the encapsulated soft tissue [30]. A pre-study FE convergence analysis was carried out on the heel of the foot focused on the calcaneus as this is where the highest and most spatially varying stresses occurred in gait simulations (Fig. 2). Tetrahedral mesh resolutions from 1 to 9 mm (in steps of 2) were simulated with the von Mises stress at the bone center averaged. Consequently, a 3-mm mesh density was chosen for simulation efficiency of 66 models as it is clear that a 3-mm mesh size is converged with von Mises stress changing by less than 2% between 1 and 3 mm. In contrast, von Mises stress changed by more than 10% between 3 and 5 mm and larger resolutions. Also, the difference between 0.51 and 0.52 MPa was within the tolerance of errors attributed to boundary conditions in the finite element model. Therefore, the mesh size for bony structure and encapsulated soft tissue was set as 3 mm, and it is also supported by a previous study [31]. The solid models were subsequently imported and assembled in the FE software Abaqus 2022 (Dassault Systems Simulia Company, Providence, RI, USA). Five plantar fasciae were built through connecting the insertion points with connector elements based on an anatomy atlas [12].

All materials assigned in the FE models were idealized as being homogeneous, isotropic, and linearly elastic, which are listed in Table 2. The Young’s modulus of bone and soft tissue was assigned as 7300 MPa and 1.15 MPa, respectively, while Poisson’s ratio was 0.3 for bone and 0.49 for soft tissue [30, 32]. The Young’s modulus of plantar fascia was assigned as 350 MPa, and Poisson’s ratio was set as 0.4 [12]. The same material properties were utilized for all three groups.

**Table 2:** The element types and material properties of the components used in finite elements models

Component	Topology structure	Formulation	Young’s modulus *E* (MPa)	Poisson’s ratio *v*
Bone [30]	3D-Tetrahedra	Linear	7300	0.3
Soft tissue [32]	3D-Tetrahedra	Linear	1.15	0.49
Plantar fascia [12]	1D	Connector	350	0.4

### Boundary and loading conditions

**Fig. 3:**
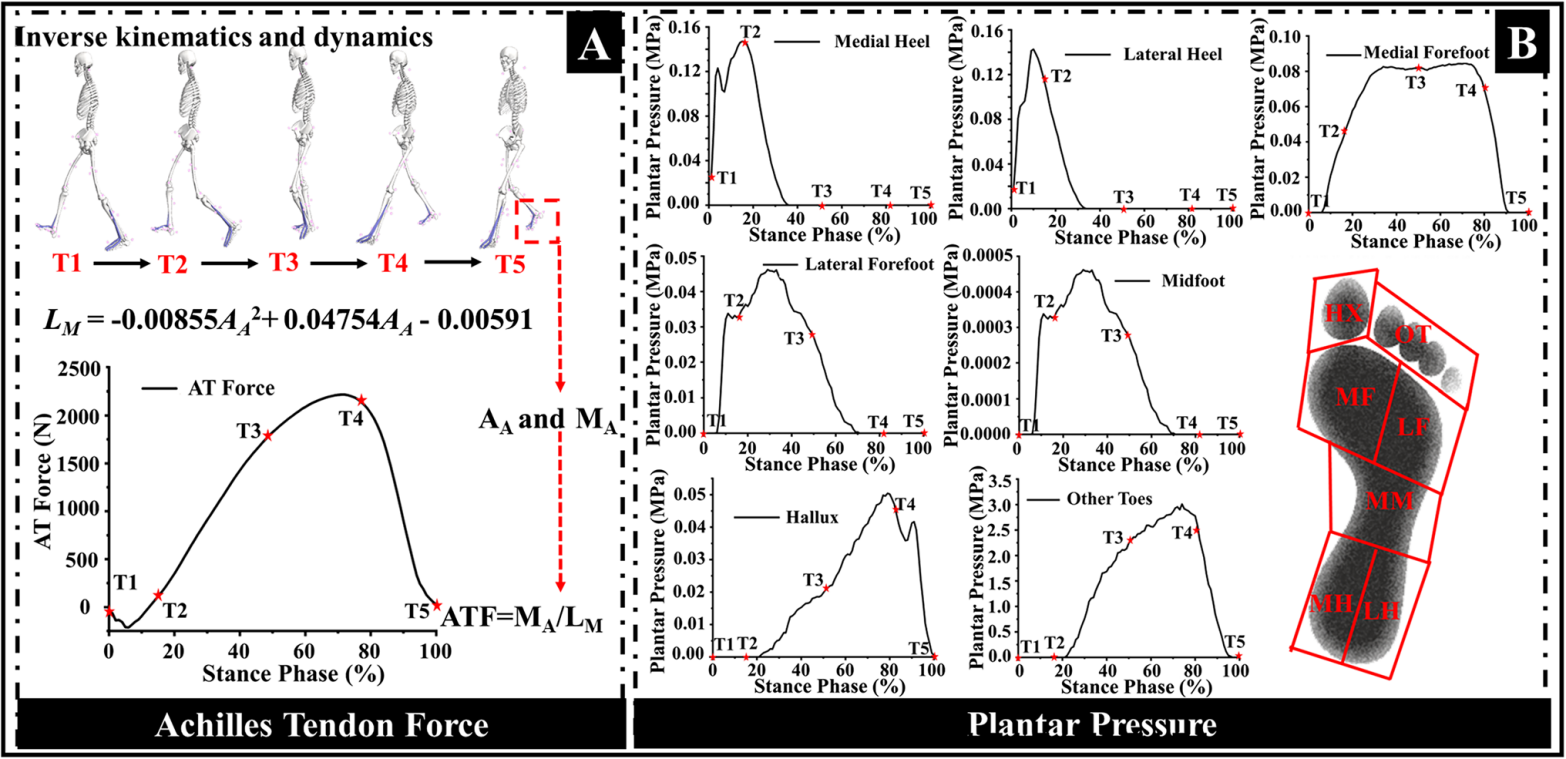
Acquisition of boundary loading conditions for finite element simulation. **A** Achilles tendon force calculation and output, and **B** regional plantar pressure definition and acquisition. A_A_ and M_A_ mean the ankle angle and ankle moment in the sagittal plane, respectively. L_M_ means the moment arm

As is shown in Fig. 1B, the proximal surfaces of the tibia, fibula, and encapsulated soft tissue were fixed in all directions. Based on our previous studies [27, 28] which synchronously conducted both three-dimensional gait analysis and foot pressure capture during the cutting maneuver, the boundary and loading conditions were accordingly determined. Joint kinetics and kinematics and plantar pressure during the stance phase of the cutting task were further processed to obtain Achilles tendon forces (ATF) (Fig. 3A) and regional plantar pressure (Fig. 3B) to drive the FE models. Plantar pressure was divided into 7 foot regions and mapped to the plantar of the FE model, including hallux, other toes, medial forefoot, lateral forefoot, midfoot, medial rearfoot, and lateral rearfoot. Ankle angle (A_A_, units: rad) was defined as the angle between the shank and the foot in the sagittal plane. Moment arm of the Achilles tendon was then estimated via the Eq. (1) which was based on the polynomial algorithm and Achilles tendon in vivo imaging data of previous studies [33, 34]. Finally, ATF were calculated by dividing ankle joint moment with moment arm (*L*_*M*_) [35] and were assigned to the superior surface of the calcaneus.

1 $${L}_{M}=-0.00855{{\text{A}}_{\text{A}}}^{2}+0.04754{\text{A}}_{\text{A}-}0.00591$$

To briefly reveal the bone stress during the stance phase of the cutting task, five key time points were selected. These included initial contact (T1), loading response (T2), mid-stance (T3), terminal stance (T4), and pre-swing (T5) [36]. Regional plantar pressure was mapped onto the corresponding foot plantar mesh of the FE models and combined with ATF to drive the FE models. Therefore, variables including ATF, regional plantar pressure, and foot geometry are different across the three groups of the FE models. The same boundary and loading condition framework were utilized for all 66 FE models. The bone structures and encapsulated soft tissue were tied together in Abaqus to prevent movement.

### Model evaluation

FE models were evaluated by comparing the peak von Mises stress in the second metatarsal at both the impact peak instant and mid-stance instant, consistent with other dynamic FE studies [30, 34], in part due to the limited literature available on FE models in cutting tasks and for chronic ankle instability. Yang et al. [32] analyzed bone stress at the impact peak during forefoot running. Yan et al. [37] compared von Mises stress in the second metatarsal bone during midstance.”

### Data analysis

The peak and mean von Mises stresses of different bones and soft tissue were obtained from the simulation. Furthermore, due to the characteristics of LAS and cutting task, the peak von Mises stresses of five different regions of soft tissue were also acquired from the FE simulation, which includes (1) soft tissue between the tibia and talus, (2) soft tissue between the fibula and talus, (3) soft tissue around the calcaneus, (4) soft tissue around 1st metatarsal, and (5) soft tissue around 5th metatarsal. Before comparing the differences between CAI, coper, and control groups, normality of the data was test via the Shapiro-Wilks test. One-way analysis of variance (ANOVA) or Kruskal–Wallis ANOVA was performed to evaluate the differences among three groups based on whether the data were normally distributed. All statistical analyses were conducted using Origin2018 (OriginLab, Northampton, USA). The alpha level of *p* ≤ 0.05 was set for all statistical tests.

## Results

### Results of model evaluation

The peak von Mises stress in the 2nd metatarsal in the current study at the loading response instant was 10.36 ± 5.30 MPa in CAI, 8.47 ± 4.05 MPa in copers, and 10.60 ± 6.68 MPa in the control group, which was within the range of the FE analysis results (9–12 MPa) in a previous study with the same time instant and loading conditions [32]. At the midstance phase, the peak von Mises stress in the 2nd metatarsal in this study was 21.88 ± 8.87 MPa in CAI, 17.53 ± 7.55 MPa in copers, and 19.82 ± 7.53 MPa in the control group, which was close to the FE results of 26 MPa in previous research [37].

### Comparisons of peak von Mises stress in soft tissue

**Fig. 4:**
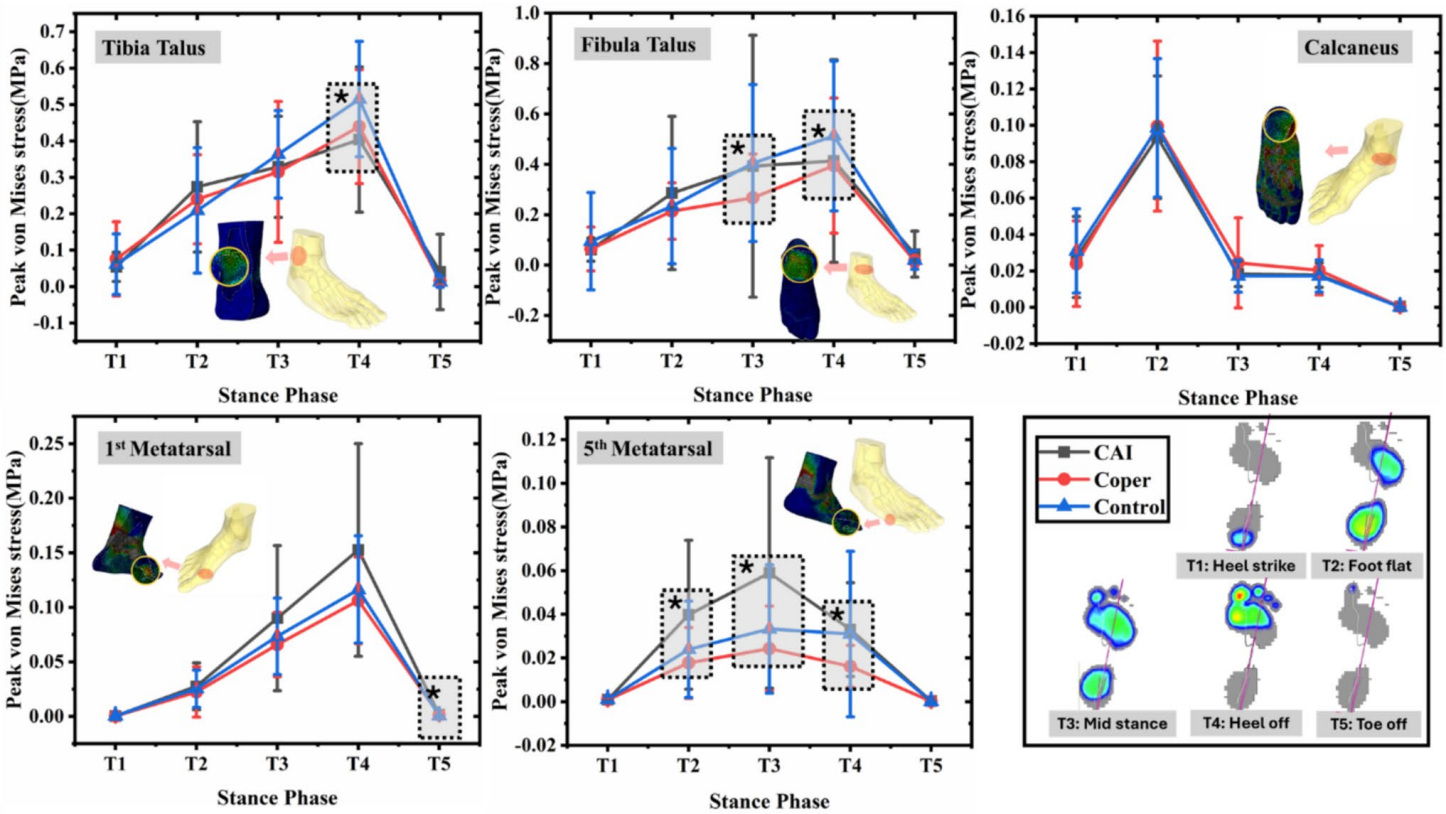
Comparisons of peak von Mises stress in five different regions of soft tissue among three groups during the stance phase of the cutting task

Peak von Mises stress in five soft tissue regions during stance phase of the cutting maneuver is revealed in Fig. 4. For the soft tissue around the calcaneus, no significant differences were observed at the five time points during stance. For the soft tissue between the talus and tibia, a group difference was found at heel-off (T4), where the control group had higher peak von Mises stress compared to CAI individuals (*P* = 0.021). Peak von Mises stress in the soft tissue between the fibula and talus at mid-stance (T3) and heel-off (T4) was significantly different among the groups, where control participants had higher stress compared to copers at mid-stance (T3) (*P* = 0.045), and to CAI at heel-off (T4) (*P* = 0.045). Peak von Mises stress of the soft tissue around the 1st metatarsal in the CAI group was significantly greater than that in the control group at toe-off (T5) (*P* = 0.034). Furthermore, significant differences were observed in the soft tissue around the 5th metatarsal, where CAI individuals exhibited higher peak von Mises stress compared to copers at foot flat (T2) (*P* = 0.003), mid-stance (T3) (*P* = 0.002), and heel-off (T4) (*P* = 0.005). CAI also had higher peak von Mises stress compared to healthy individuals at foot flat (T2) (*P* = 0.049).

### Comparison of peak von Mises stress in bone structures

Peak von Mises stress observed in the foot bones is presented in Table 3 with significant differences among the three groups exhibited at time points of foot flat (T2), mid-stance (T3), and heel-off (T4). At the time point of the foot flat (T2), CAI had significantly higher stress compared to copers in the navicular (*P* = 0.046) and intermediate cuneiform (*P* = 0.005). At mid-stance (T3), peak stress of CAI was significantly higher compared to copers in the navicular (*P* = 0.014), medial cuneiform (*P* = 0.029), intermediate cuneiform (*P* = 0.003), lateral cuneiform (*P* = 0.003), 3rd metatarsal (*P* = 0.018), 4th metatarsal (*P* = 0.044), and phalanges (*P* = 0.040). At the heel-off (T4), the control group had higher peak von Mises stress compared to CAI individuals in the talus (*P* = 0.022). However, CAI showed higher stress compared to controls in the intermediate cuneiform (*P* = 0.009). Furthermore, CAI individuals had higher peak von Mises stress compared to copers in the navicular (*P* = 0.001), media cuneiform (*P* = 0.011), intermediate cuneiform (*P* < 0.001), and lateral cuneiform (*P* = 0.029).

**Table 3:** Peak von Mises stresses in the bones of the foot–ankle complex during the stance phase of the cutting task

Time point	Bone	Peak von Mises (MPa)			ANOVA (KWANOVA)	Post hoc
		CAI	Coper	Control		
T2	Tibia	15.17 (17.43)	9.87 (4.64)	10.90 (7.76)	0.725	/
	Fibula	17.60 (10.14)	19.82 (10.18)	17.17 (10.04)	0.321	/
	Talus	10.94 (12.09)	11.70 (9.22)	9.95 (8.23)	0.517	/
	Calcaneus	29.62 (27.89)	32.25 (19.39)	29.15 (16.65)	0.580	/
	Navicular	9.73 (16.69)	5.40 (6.00)	5.30 (3.19)	0.0516^*^	CAI > coper (*P* = 0.046)
	MC	13.22 (11.73)	7.20 (4.93)	11.55 (9.94)	0.110	/
	IMC	19.84 (13.59)	10.09 (6.37)	14.24 (6.59)	0.006^*^	CAI > coper (*P* = 0.005)
	LC	15.14 (10.48)	10.55 (6.94)	11.47 (8.68)	0.473	/
	Cuboid	12.03 (10.93)	9.27 (7.05)	14.69 (12.96)	0.313	/
	1st metatarsal	11.19 (7.99)	8.32 (4.39)	9.39 (5.12)	0.460	/
	2nd metatarsal	10.36 (5.30)	8.47 (4.05)	10.60 (6.68)	0.564	/
	3rd metatarsal	15.81 (7.95)	11.68 (4.86)	14.27 (11.18)	0.287	/
	4th metatarsal	18.10 (8.28)	14.61 (6.61)	16.26 (6.94)	0.490	/
	5th metatarsal	14.59 (7.12)	11.07 (5.56)	13.44 (6.14)	0.176	/
	Phalanges	6.24 (4.04)	4.07 (2.22)	5.87 (3.23)	0.128	/
T3	Tibia	16.02 (5.97)	14.42 (11.85)	17.67 (9.77)	0.096	/
	Fibula	22.90 (8.21)	22.09 (8.98)	30.95 (17.72)	0.128	/
	Talus	12.64 (5.98)	18.59 (26.38)	17.97 (11.92)	0.169	/
	Calcaneus	88.07 (41.02)	80.53 (39.09)	93.08 (31.59)	0.311	/
	Navicular	11.08 (11.85)	6.65 (5.92)	6.80 (3.84)	0.018^*^	CAI > coper (*P* = 0.014)
	MC	36.98 (26.41)	21.62 (18.44)	26.71 (18.25)	0.034^*^	CAI > coper (*P* = 0.029)
	IMC	37.20 (21.10)	21.75 (17.98)	25.48 (12.94)	0.004^*^	CAI > coper (*P* = 0.003)
	LC	20.15 (9.81)	11.33 (7.36)	11.99 (5.33)	0.002^*^	CAI > coper (*P* = 0.003); CAI > control (*P* = 0.013)
	Cuboid	19.30 (14.02)	13.37 (8.78)	17.02 (10.53)	0.371	/
	1st metatarsal	29.04 (14.94)	23.12 (11.74)	23.36 (8.25)	0.186	/
	2nd metatarsal	21.88 (8.87)	17.53 (7.55)	19.82 (7.53)	0.237	/
	3rd metatarsal	28.20 (12.01)	20.14 (7.50)	21.93 (8.34)	0.017^*^	CAI > coper (*P* = 0.018)
	4th metatarsal	28.80 (10.86)	21.90 (9.05)	23.69 (7.84)	0.046^*^	CAI > coper (*P* = 0.044)
	5th metatarsal	18.74 (9.64)	12.91 (6.15)	15.19 (7.44)	0.118	/
	Phalanges	15.80 (7.76)	9.81 (5.06)	11.90 (6.17)	0.043^*^	CAI > coper (*P* = 0.040)
T4	Tibia	20.24 (11.92)	24.16 (23.89)	34.89 (34.89)	0.293	/
	Fibula	30.46 (21.97)	34.92 (26.20)	42.07 (27.27)	0.155	/
	Talus	18.01 (13.98)	24.52 (26.01)	48.85 (73.47)	0.025^*^	Control > CAI (*P* = 0.022)
	Calcaneus	116.42 (55.94)	112.70 (41.64)	122.89 (44.92)	0.560	/
	Navicular	10.61 (4.95)	6.16 (2.27)	9.29 (9.69)	0.002^*^	CAI > coper (*P* = 0.001)
	MC	53.72 (28.86)	28.56 (18.91)	36.94 (24.73)	0.014^*^	CAI > coper (*P* = 0.011)
	IMC	51.10 (20.00)	29.62 (18.03)	35.11 (13.55)	< 0.001^*^	CAI > coper (*P* < 0.001); CAI > control (*P* = 0.009)
	LC	24.45 (14.09)	15.76 (8.50)	17.45 (8.77)	0.026^*^	CAI > coper (*P* = 0.029)
	Cuboid	18.20 (14.20)	12.11 (7.52)	16.34 (9.48)	0.411	/
	1st metatarsal	43.35 (22.07)	32.53 (15.91)	32.22 (11.72)	0.056	/
	2nd metatarsal	26.11 (11.73)	21.46 (7.93)	24.63 (9.61)	0.286	/
	3rd metatarsal	27.97 (14.00)	20.45 (7.09)	23.44 (11.65)	0.139	/
	4th metatarsal	23.53 (7.80)	19.54 (6.81)	22.12 (8.63)	0.233	/
	5th metatarsal	10.92 (4.53)	9.87 (5.40)	10.89 (5.67)	0.711	/
	Phalanges	28.97 (17.46)	22.09 (11.95)	24.05 (11.94)	0.253	/

*CAI*, chronic ankle instability; *KWANOVA*, Kruskal–Wallis ANOVA; *T2*, time point of foot flat; *T3*, time point of mid stance; *T4*, time point of heel off; *MC*, media cuneiform; *IMC*, intermediate cuneiform; *LC*, lateral cuneiform; ^*^indicates significant differences between groups

### Comparison of mean von Mises stress in bone structures

Significant group differences in mean von Mises stress were found during foot flat (T2), mid-stance (T3), and heel-off (T4) (Table 4). At foot flat (T2), mean von Mises stress in CAI was significantly greater than the corresponding stress in copers in the 5th metatarsal (*P* = 0.034) and phalanges (*P* = 0.024). At mid-stance (T3), the control group had higher stress compared to copers in the fibula (*P* = 0.022) and calcaneus (*P* = 0.019). CAI individuals showed higher mean von Mises stress compared to copers in the navicular (*P* = 0.017), intermediate cuneiform (*P* = 0.009), lateral cuneiform (*P* = 0.006), 3rd metatarsal (*P* = 0.006), 4th metatarsal (*P* = 0.008), and 5th metatarsal (*P* = 0.037). Further, mean von Mises stress of CAI was higher than the control group in the 3rd metatarsal (*P* = 0.033). At heel-off (T4), the control group had higher stress compared to CAI in the fibula (*P* = 0.024), but CAI had higher stress compared to the control group in the lateral cuneiform (*P* = 0.049). Furthermore, CAI individuals had higher mean von Mises stress compared to copers in the navicular (*P* = 0.012), medial cuneiform (*P* = 0.039), intermediate cuneiform (*P* = 0.001), and lateral cuneiform (*P* = 0.005).

**Table 4:** Mean von Mises stresses in the bones of the foot–ankle complex during the stance phase of the cutting task

Time point	Bone	Mean von Mises (MPa)			ANOVA (KWANOVA)	Post hoc
		CAI	Coper	Control		
T2	Tibia	2.32 (1.44)	1.8 (0.70)	1.83 (0.98)	0.398	/
	Fibula	4.61 (2.25)	4.20 (1.63)	4.03 (1.08)	0.862	/
	Talus	1.66 (0.79)	1.47 (0.54)	1.41 (0.59)	0.371	/
	Calcaneus	1.95 (0.85)	1.87 (0.77)	1.94 (0.78)	0.996	/
	Navicular	1.17 (0.44)	0.86 (0.34)	1.07 (0.54)	0.096	/
	MC	0.78 (0.33)	0.60 (0.23)	0.77 (0.52)	0.238	/
	IMC	1.77 (0.88)	1.16 (0.55)	1.51 (0.83)	0.071	/
	LC	1.54 (0.68)	1.13 (0.55)	1.28 (0.54)	0.077	/
	Cuboid	1.75 (0.92)	1.42 (0.73)	1.90 (0.90)	0.257	/
	1st metatarsal	0.83 (0.32)	0.78 (0.34)	0.84 (0.41)	0.875	/
	2nd metatarsal	2.01 (0.86)	1.54 (0.61)	1.80 (1.00)	0.139	/
	3rd metatarsal	2.97 (1.38)	2.18 (0.92)	2.54 (1.23)	0.203	/
	4th metatarsal	3.34 (1.61)	2.50 (1.09)	2.87 (1.23)	0.261	/
	5th metatarsal	3.01 (1.55)	2.07 (0.92)	2.52 (1.09)	0.044^*^	CAI > coper (*P* = 0.034)
	Phalanges	0.62 (0.33)	0.37 (0.17)	0.60 (0.35)	0.015^*^	CAI > coper (*P* = 0.024)
T3	Tibia	2.83 (0.92)	2.38 (1.17)	2.60 (0.78)	0.311	/
	Fibula	5.86 (1.90)	5.17 (2.11)	6.80 (1.92)	0.029^*^	Control > coper (*P* = 0.022)
	Talus	2.25 (0.55)	2.13 (0.97)	2.47 (0.71)	0.338	/
	Calcaneus	3.99 (0.91)	3.46 (1.17)	4.34 (1.02)	0.024^*^	Control > coper (*P* = 0.019)
	Navicular	1.73 (0.60)	1.21 (0.56)	1.46 (0.72)	0.020^*^	CAI > coper (*P* = 0.017)
	MC	1.88 (0.65)	1.51 (0.81)	1.75 (0.90)	0.097	/
	IMC	3.26 (1.17)	2.18 (1.28)	2.55 (1.11)	0.011^*^	CAI > coper (*P* = 0.009)
	LC	2.18 (0.90)	1.42 (0.77)	1.62 (0.57)	0.008^*^	CAI > coper (*P* = 0.006)
	Cuboid	2.64 (1.04)	2.06 (1.22)	2.47 (0.95)	0.069	/
	1st metatarsal	2.04 (0.62)	1.86 (0.70)	1.91 (0.55)	0.642	/
	2nd metatarsal	3.69 (1.25)	2.76 (1.05)	3.11 (1.30)	0.060	/
	3rd metatarsal	4.88 (1.89)	3.45 (1.31)	3.74 (1.11)	0.005^*^	CAI > coper (*P* = 0.006); CAI > control (*P* = 0.033)
	4th metatarsal	5.12 (2.01)	3.66 (1.38)	4.01 (1.21)	0.008^*^	CAI > coper (*P* = 0.008)
	5th metatarsal	3.79 (2.12)	2.37 (1.01)	2.67 (0.97)	0.036^*^	CAI > coper (*P* = 0.037)
	Phalanges	1.54 (0.66)	1.19 (0.63)	1.43 (0.52)	0.284	/
T4	Tibia	3.13 (1.07)	2.81 (1.14)	3.38 (1.32)	0.314	/
	Fibula	6.05 (2.43)	6.38 (2.95)	8.31 (2.90)	0.018^*^	Control > CAI (*P* = 0.024)
	Talus	2.79 (0.80)	2.87 (1.16)	3.59 (1.66)	0.224	/
	Calcaneus	4.90 (1.19)	4.58 (1.50)	5.47 (1.37)	0.097	/
	Navicular	1.89 (0.59)	1.39 (0.47)	1.75 (0.91)	0.016^*^	CAI > coper (*P* = 0.012)
	MC	2.80 (0.96)	2.11 (0.85)	2.47 (1.28)	0.039^*^	CAI > coper (*P* = 0.039)
	IMC	3.98 (1.38)	2.61 (1.12)	3.20 (1.43)	0.002^*^	CAI > coper (*P* = 0.001)
	LC	2.50 (0.92)	1.74 (0.78)	1.94 (0.59)	0.005^*^	CAI > coper (*P* = 0.005); CAI > control (*P* = 0.049)
	Cuboid	2.49 (1.07)	1.99 (1.02)	2.41 (0.91)	0.141	/
	1st metatarsal	3.03 (1.02)	2.58 (0.89)	2.71 (1.02)	0.224	/
	2nd metatarsal	4.38 (1.56)	3.47 (1.29)	3.96 (1.89)	0.189	/
	3rd metatarsal	4.50 (1.61)	3.49 (1.21)	3.81 (1.37)	0.059	/
	4th metatarsal	4.26 (1.39)	3.45 (1.17)	3.87 (1.40)	0.080	/
	5th metatarsal	2.20 (0.77)	1.84 (0.78)	2.14 (1.04)	0.216	/
	Phalanges	3.15 (1.74)	2.31 (1.09)	2.87 (1.44)	0.290	/

*CAI*, chronic ankle instability; *KWANOVA*, Kruskal–Wallis ANOVA; *T2*, time point of foot flat; *T3*, time point of mid stance; *T4*, time point of heel off; *MC*, media cuneiform; *IMC*, intermediate cuneiform; *LC*, lateral cuneiform; ^*^indicates significant differences between groups

## Discussion

This study developed sixty-six personalized FE foot models and utilized computation simulation to investigate the internal foot mechanics of CAI, copers, and controls. Specifically, this study focused on alterations in internal bone and soft tissue foot stress during the stance phase of a cutting task. It was found that group differences were exhibited in both soft tissue and foot bone stress at foot flat, mid-stance, and heel-off phases. In general, healthy individuals showed higher stress in soft tissue around the ankle joint and relevant foot bones compared to CAI in midstance and heel-off phases. Contrarily, CAI had higher stress in soft tissue around the 1st metatarsal and 5th metatarsal at toe-off and foot flat phases, separately. Moreover, CAI individuals had higher stress in the lateral cuneiform at mid-stance and intermediate cuneiform at heel-off compared to controls. More significant differences in bone stress were observed between CAI and copers, where CAI individuals had higher stress in the midfoot bones and lateral forefoot bones at foot flat, mid-stance, and heel-off phases.

Consistent with our hypothesis, group differences were observed in soft tissue around the ankle joint and talus at the loading response and push-off phases of stance. No significant differences were found among the three groups at the initial contact phase, which might be due to geometrical features of the ankle joint. A dorsiflexed position at heel strike increases the contact area of the talus, fibula, and tibia and hence stabilizes the ankle joint [19]. In the current study, it was found that CAI showed higher stress in both soft tissue and bones in the lateral forefoot region including soft tissue around the 3rd and 5th metatarsals and 4th metatarsal bones at mid-stance and push-off phases. Based on previous studies [3, 38], lateral ankle ligaments influence the shift of the center of pressure (COP) trajectory. The anterior talofibular ligament and calcaneofibular ligaments influence the COP trajectory at toe-off and the entire stance phase, respectively. While the posterior talofibular ligament affects the COP trajectory at heel strike. Repetitive LAS results in structural damage on the lateral ankle ligaments, primarily on the anterior talofibular and calcaneofibular ligaments [1, 27], and this may in part explain the higher stress in CAI that was observed at the push-off phase. The increase of stress in both soft tissue and bones in the lateral forefoot region may be due to the COP trajectory of CAI patients shifting anterolaterally [38]. The increased localized stress in the lateral forefoot region also raises further concerns and may be an etiologic factor in osteoarthritis development [39].

Interestingly, healthy individuals were found to have higher stress in soft tissue around the talus and bones during the push-off stance in this study. Although CAI individuals may show increased joint contact stress and accordingly develop ankle osteoarthritis [39, 40], ankle osteoarthritis is not prevalent in all CAI patients with a 43% incidence [41, 42]. Besides, previous studies reported no differences in cartilage morphology for college-aged CAI individuals and age-matched healthy individuals [43, 44]. All participants in the current study were recruited from a college population, which may explain why no higher stress around the talus was observed in CAI and copers. In contrast, both CAI individuals and copers presented lower stress compared to healthy controls, which might be a compensatory mechanism adopted. By minimizing stress around the ankle joint, both CAI individuals and copers may be minimizing pain and reducing potential damage at the ankle joint while restoring stability of the ankle [45]. Furthermore, considering the anatomical shape of the talus, which is wide in the front and narrow in the back, during push-off, the ankle is in a relatively unstable condition, and differences between groups are thus more prominent at this stage [45].

Three cuneiforms (medial, intermediate, lateral) function as the stabilizing structure within the medial region of the foot. Together with the cuboid proximally, and the base of the five metatarsal heads distally, they compose the Lisfranc joint (where the metatarsal bones connect to the rest of your foot and links the midfoot and forefoot), which plays a role in transferring the forces generated by calf muscles to the distal region of the foot [46, 47]. The Lisfranc joint remains mobile at initial contact then acts as a rigid lever during push-off [48]. Higher stress in the three cuneiforms was found in CAI individuals compared to healthy controls and copers at mid-stance and heel-off phases. Combined with lower stress around the talus and higher stress in the lateral forefoot, it appears that CAI individuals adopt a protective strategy via transferring joint forces to the anterolateral foot during mid-stance and push-off phases. Owing to bony anatomy and strong attachment of the ligaments, the Lisfranc joint is relatively stable and the injuries in that region are relatively rare [48]. However, one still needs to be cautious about stress increase in the cuneiforms since axial loading or twisting on the plantarflexed foot would be an indirect mechanism to Lisfranc injury [48].

Although this is the first study using personalized FE models to help better understand foot bone and tissue stress in CAI during a cutting task, several limitations should be considered when interpreting the results of this study. A major consideration is that no ligaments and cartilage were developed in the current FE study. The importance of lateral ankle ligaments, particularly the calcaneofibular ligament and the anterior talofibular ligament, was highlighted in previous studies [3, 27]. However, the primary boundary condition for mechanics in this study was plantar pressure, joint contact, and Achilles tendon force. Furthermore, given the fact that LAS might lead to structural damage in ligamentous tissue [49], it is still unclear whether the material properties would be altered for lateral ankle ligaments when modeling FE models for CAI and copers. To provide more valuable findings, a consideration of ligamentous tissue property remodeling among these three groups would add further value to the findings in this study. Our model used 7 regional plantar pressure zones from experiments that were mapped to the plantar of the FE models as boundary and loading conditions; however, FE results could be more precise if more detailed regional plantar pressure were adopted. Finally, only males were included in the current study because of a relatively higher risk of suffering a subsequent LAS compared to females [27, 50]. Therefore, our study findings will likely have different tissue and bone stress outcomes in a female population [50].

## Conclusion

In summary, this study developed sixty-six personalized FE models of CAI, copers, and healthy controls to evaluate bone and soft tissue stress in response to a cutting task. This is the first study to evaluate such a large cohort computationally using FE analysis.

Key simulation findings include the following.

- Group differences of mechanical stress in both soft tissue and bone structures were mainly presented at the push-off phase.
- Healthy individuals had significantly higher stress on the talus bone and soft tissue around the talus, whereas CAI presented higher stress on the cuneiforms and bones of the lateral forefoot.
- Implications from the results: CAI individuals appear to adopt a protective strategy by transferring joint force to the lateral forefoot at the heel-off phase, and this lowers stress around the talus, which may be a strategy to minimize ankle pain and avoid joint damage.
- These findings highlight that internal bone and tissue stress needs to be considered as part of rehabilitation strategy with possible implications for osteoarthritis of the lateral forefoot region due to higher stresses in CAI.

## Supplementary Information

Below is the link to the electronic supplementary material.

Supplementary file1 (DOCX 27 KB)
