# Supplementary material for: Foot tissue stress in chronic ankle instability during the stance phase of cutting
Source: Med Biol Eng Comput. 2025 Jan 15;63(5):1507–19. doi: 10.1007/s11517-024-03276-9 (PMC12064455; doi:10.1007/s11517-024-03276-9)
Supplement: Supplementary file 1 — Supplementary file1 (DOCX 27 KB) [file 11517_2024_3276_MOESM1_ESM.docx]

***Von Mises stresses in the bones during the stance phase of the cutting task.***

Predicted peak von Mises stresses and mean von Mises stresses in the foot bones among three groups at the time points of the stance phase of the cutting tasks are presented in Table S1 and Table S2, separately. No significant differences among these three groups were observed at the time point of initial contact as well as toe-off.

******Insert Table S1 here******

******Insert Table S2 here******

**Table S1**. Peak von Mises stresses in the bones of the foot-ankle complex during the stance phase of the cutting task.

| **Time point** | **Bone** | **Peak von Mises (MPa)** | | | **ANOVA**  **(KWANOVA)** | **Post hoc** |
| --- | --- | --- | --- | --- | --- | --- |
|  |  | **CAI** | **Coper** | **Control** |  |  |
| T1 | Tibia | 2.14(2.90) | 2.35(3.11) | 2.60 (4.00) | 0.709 | / |
|  | Fibula | 2.86 (2.73) | 4.93 (7.85) | 4.02(4.71) | 0.991 | / |
|  | Talus | 1.80(1.97) | 3.45(6.00) | 3.42(5.39) | 0.404 | / |
|  | Calcaneus | 8.33 (7.15) | 9.98 (13.15) | 9.37(8.86) | 0.756 | / |
|  | Navicular | 0.87(1.08) | 0.88 (1.28) | 0.88(0.96) | 0.501 | / |
|  | MC | 0.83(0.62) | 0.97(1.18) | 1.05 (1.22) | 0.638 | / |
|  | IMC | 0.94 (0.63) | 0.87(0.96) | 1.26 (1.22) | 0.197 | / |
|  | LC | 0.78 (0.78) | 0.68(0.60) | 0.75 (0.67) | 0.710 | / |
|  | Cuboid | 0.70(0.79) | 0.56(0.58) | 1.04 (1.56) | 0.309 | / |
|  | 1^ST^ Metatarsal | 0.35 (0.37) | 0.56 (0.73) | 0.39(0.64) | 0.288 | / |
|  | 2^nd^ Metatarsal | 0.25 (0.17) | 0.39 (0.44) | 0.31 (0.40) | 0.728 | / |
|  | 3^rd^ Metatarsal | 0.31 (0.26) | 0.40 (0.45) | 0.39(0.49) | 0.903 | / |
|  | 4^th^ Metatarsal | 0.42(0.30) | 0.56(0.65) | 0.44(0.55) | 0.688 | / |
|  | 5^th^ Metatarsal | 0.70 (0.64) | 0.53(0.52) | 0.81(0.71) | 0.370 | / |
|  | Phalanges | 0.15 (0.12) | 0.16(0.16) | 0.24 (0.24) | 0.071 | / |
| T5 | Tibia | 1.41 (3.46) | 0.46 (0.37) | 0.48(0.51) | 0.787 | / |
|  | Fibula | 1.72 (3.71) | 0.89 (0.84) | 0.92 (1.28) | 0.344 | / |
|  | Talus | 1.14 (2.18) | 0.56(0.41) | 0.53(0.53) | 0.476 | / |
|  | Calcaneus | 6.36 (17.40) | 1.74(1.53) | 1.90 (2.22) | 0.790 | / |
|  | Navicular | 1.13(4.15) | 0.18 (0.33) | 0.14 (0.23) | 0.551 | / |
|  | MC | 0.53(0.77) | 0.37 (0.68) | 0.19 (0.13) | 0.242 | / |
|  | IMC | 0.66 (1.19) | 0.29(0.40) | 0.20(0.16) | 0.222 | / |
|  | LC | 0.28(0.41) | 0.17 (0.29) | 0.09(0.08) | 0.106 | / |
|  | Cuboid | 0.39(1.10) | 0.10 (0.09) | 0.18 (0.31) | 0.887 | / |
|  | 1^ST^ Metatarsal | 0.45 (0.66) | 0.26 (0.39) | 0.15 (0.10) | 0.117 | / |
|  | 2^nd^ Metatarsal | 0.29(0.43) | 0.18 (0.26) | 0.10(0.06) | 0.371 | / |
|  | 3^rd^ Metatarsal | 0.29 (0.52) | 0.14(0.18) | 0.10 (0.07) | 0.338 | / |
|  | 4^th^ Metatarsal | 0.36(0.76) | 0.14(0.15) | 0.12 (0.11) | 0.601 | / |
|  | 5^th^ Metatarsal | 0.19 (0.42) | 0.07 (0.08) | 0.06 (0.06) | 0.522 | / |
|  | Phalanges | 0.47 (0.74) | 0.40 (0.55) | 0.23 (0.28) | 0.132 | / |

CAI, chronic ankle instability; KWANOVA, Kruskal-Wallis ANOVA; T1, time point of initial contact; T5, time point of toe off; MC, media cuneiform; IMC, intermediate cuneiform; LC, lateral cuneiform.

**Table S2**. Mean von Mises stresses in the bones of the foot-ankle complex during the stance phase of the cutting task.

| **Time point** | **Bone** | **Mean von Mises (MPa)** | | | **ANOVA**  **(KWANOVA)** | **Post hoc** |
| --- | --- | --- | --- | --- | --- | --- |
|  |  | **CAI** | **Coper** | **Control** |  |  |
| T1 | Tibia | 0.37 (0.29) | 0.50(0.69) | 0.43 (0.60) | 0.674 | / |
|  | Fibula | 0.74 (0.60) | 0.99(1.40) | 0.87 (1.04) | 0.398 | / |
|  | Talus | 0.29 (0.23) | 0.39 (0.51) | 0.35(0.40) | 0.811 | / |
|  | Calcaneus | 0.38 (0.24) | 0.47(0.59) | 0.50 (0.55) | 0.473 | / |
|  | Navicular | 0.12(0.07) | 0.13(0.12) | 0.14 (0.11) | 0.552 | / |
|  | MC | 0.05 (0.03) | 0.07 (0.07) | 0.06 (0.07) | 0.934 | / |
|  | IMC | 0.09(0.04) | 0.10 (0.11) | 0.10 (0.09) | 0.842 | / |
|  | LC | 0.09(0.05) | 0.08 (0.07) | 0.10(0.07) | 0.633 | / |
|  | Cuboid | 0.10 (0.07) | 0.10 (0.09) | 0.15 (0.16) | 0.300 | / |
|  | 1^ST^ Metatarsal | 0.04 (0.03) | 0.07 (0.09) | 0.05 (0.07) | 0.432 | / |
|  | 2^nd^ Metatarsal | 0.07 (0.05) | 0.10 (0.12) | 0.08 (0.11) | 0.691 | / |
|  | 3^rd^ Metatarsal | 0.07 (0.05) | 0.09 (0.10) | 0.08 (0.09) | 0.885 | / |
|  | 4^th^ Metatarsal | 0.10 (0.06) | 0.12 (0.14) | 0.10 (0.10) | 0.667 | / |
|  | 5^th^ Metatarsal | 0.13 (0.09) | 0.12(0.12) | 0.15 (0.12) | 0.269 | / |
|  | Phalanges | 0.01 (0.01) | 0.02 (0.02) | 0.02 (0.02) | 0.373 | / |
| T5 | Tibia | 0.29(0.73) | 0.11(0.09) | 0.09 (0.11) | 0.463 | / |
|  | Fibula | 0.48 (1.15) | 0.18(0.15) | 0.19(0.21) | 0.628 | / |
|  | Talus | 0.19(0.44) | 0.07 (0.05) | 0.07 (0.08) | 0.561 | / |
|  | Calcaneus | 0.25(0.58) | 0.08(0.05) | 0.09 (0.10) | 0.875 | / |
|  | Navicular | 0.05(0.10) | 0.02 (0.02) | 0.02(0.02) | 0.652 | / |
|  | MC | 0.04(0.06) | 0.03(0.03) | 0.01 (0.01) | 0.482 | / |
|  | IMC | 0.05 (0.10) | 0.03 (0.04) | 0.02(0.02) | 0.467 | / |
|  | LC | 0.04(0.06) | 0.02(0.03) | 0.01 (0.01) | 0.176 | / |
|  | Cuboid | 0.04 (0.10) | 0.02(0.02) | 0.02 (0.02) | 0.915 | / |
|  | 1^ST^ Metatarsal | 0.05(0.07) | 0.03(0.04) | 0.02(0.01) | 0.339 | / |
|  | 2^nd^ Metatarsal | 0.07(0.117) | 0.05 (0.06) | 0.02 (0.02) | 0.405 | / |
|  | 3^rd^ Metatarsal | 0.06(0.10) | 0.03(0.03) | 0.02(0.02) | 0.278 | / |
|  | 4^th^ Metatarsal | 0.07 (0.14) | 0.03 (0.03) | 0.02 (0.02) | 0.395 | / |
|  | 5^th^ Metatarsal | 0.04 (0.09) | 0.02(0.02) | 0.02(0.01) | 0.716 | / |
|  | Phalanges | 0.04 (0.06) | 0.04(0.06) | 0.02(0.01) | 0.069 | / |

CAI, chronic ankle instability; KWANOVA, Kruskal-Wallis ANOVA; T1, time point of initial contact; T5, time point of toe off; MC, media cuneiform; IMC, intermediate cuneiform; LC, lateral cuneiform.
